# Longitudinal Outcomes Following Surgical Repair of Primary Mitral Regurgitation

**DOI:** 10.3390/jcdd10030095

**Published:** 2023-02-23

**Authors:** Yuan Qiu, Hiroki Takaya, Kay Maeda, David Messika-Zeitoun, Marc Ruel, Thierry Mesana, Vincent Chan

**Affiliations:** Division of Cardiac Surgery, University of Ottawa Heart Institute, Ottawa, ON K1Y 4W7, Canada

**Keywords:** mitral valve repair, outcomes, longitudinal, long-term, minimally invasive, robotic, transcatheter

## Abstract

Degenerative mitral valve (MV) disease is the most common cause of organic mitral regurgitation (MR) in developed countries. Surgical mitral valve repair is the gold standard treatment for primary MR. Surgical mitral valve repair is associated with excellent outcomes in terms of survival and freedom from recurrent MR. As well, innovations in surgical repair techniques, including thoracoscopically and robotically assisted approaches, further reduce morbidity. Emerging catheter-based therapies may also provide advantages in select patient groups. Although the outcomes following surgical mitral valve repair are well described in the literature, longitudinal follow-up is heterogenous. Indeed, longitudinal follow-up and long-term data are vital to better advise treatment and counsel patients.

## 1. Introduction

Degenerative mitral valve (MV) disease is the most common cause of organic mitral regurgitation (MR) in developed countries [1,2]. Surgical mitral valve repair is the gold standard therapy for treating primary MR [1,3,4,5,6,7]. Refinement in techniques, in conjunction with standard annuloplasty, have led to excellent outcomes [8]. Long-term results are often reported as freedom from reoperation, which may lead to an underestimation of recurrent MR as surgery may be deferred in selected patients [8,9,10]. Few studies and centers have reported long-term outcomes following the repair of degenerative MR. Longitudinal assessment requires a dedicated infrastructure and intentional data collection. Additionally, the emergence of minimally invasive and transcatheter approaches now pose even more alternatives for treating degenerative MR [11,12,13,14,15,16,17,18]. This review highlights the need to assess longitudinal outcomes for these innovations to better counsel patients regarding different treatment options and their expected long-term outcomes.

## 2. Overview of Longitudinal Outcomes after Mitral Valve Repair

Surgical mitral valve repair provides long-term survival and morbidity benefits in patients with degenerative MR. The Society of Thoracic Surgeons (STS) adult cardiac surgery database is the world’s largest cardiac surgical database and includes data from over 1000 contributing North American centers. Badhwar et al. identified 14,604 patients who underwent isolated nonemergent primary mitral valve repair in the STS adult cardiac surgery database and linked these data to longitudinal claims data from the Centers for Medicare and Medicaid Services (CMS) [19]. More than 50% of patients were in the New York Heart Association (NYHA) class III or IV heart failure, yet the operative mortality was less than 3%. The literature often highlights the importance of expert valve disease management centers, and although relevant, this analysis demonstrates a low mortality across all STS sites, not exclusively expert mitral valve repair centers. The mean follow-up was 5.9 ± 3.9 years and the long-term survival after surgical mitral valve repair matched that of the average US population. This study highlights the safety of surgical mitral valve repair in patient subsets such as the elderly. Furthermore, early complications such as stroke and bleeding were rare. However, nuanced echocardiographic data were not available in this study.

There currently are no guidelines advising on the role of echocardiographic surveillance in asymptomatic patients after surgical mitral valve repair. It is an important adjunct in assessing valve repair failure and left ventricular remodeling. We included studies that reported long-term outcomes assessed clinically and with echocardiography to minimize reporting bias in patients who undergo postoperative echocardiography due to symptoms. Table 1 provides a summary of the studies evaluating the longitudinal outcomes of patients undergoing mitral valve repair for anterior, posterior, or bileaflet pathologies and their associated follow-up.

### 2.1. Anterior Leaflet Pathology

Anterior leaflet pathology has been reported by some to be associated with a lower rate of successful repair [19,20,21,22]. Hence, this may be a deterrent for early surgical referral in asymptomatic patients compared with patients with posterior leaflet pathologies. Gillinov et al. followed 3544 patients who were undergoing surgical mitral valve repair with either an isolated anterior leaflet (n = 307) or isolated posterior leaflet (n = 2754) procedure [22]. They followed the patients systematically at 2, 5, 10, 15, and 20 years after operation via questionnaire. Patients with anterior disease were older in age and had more pronounced changes in their cardiac structure and function, including greater left atrial enlargement, more atrial fibrillation, and reduced left ventricular function. Patients who underwent anterior leaflet repair were more likely to have 3+ to 4+ MR at follow-up compared with those patients who underwent a posterior leaflet repair, but propensity-adjusted survival was similar amongst both groups [22]. There was no long-term echocardiographic follow-up in this cohort.

Javadikasgari et al. reported a significantly increased recurrence of severe MR 10 years after repair in complex pathology patients (anterior and bileaflet) compared with simple pathology (isolated posterior leaflet). However, the 20-year survival rate for both groups were similar (62% for simple vs. 62% for complex) [20]. There was sufficient echocardiographic data to estimate valve durability to 10 years, but this follow-up with echocardiography was limited to patients who routinely returned for clinic visits [20].

Longitudinal echocardiographic data describing the outcomes following surgical mitral valve repair in patients with anterior leaflet pathology is sparsely reported. Chan et al. followed 855 patients who underwent surgical mitral valve repair caused by leaflet prolapse with echocardiographic assessments at 1 month, 3 to 6 months, and 12 months after reconstruction [10]. Beyond 1 year, patients were assessed either annually or when clinically indicated. This is the first reported large cohort study which followed patients with echocardiograms at distinct intervals postoperatively, including asymptomatic patients. Recurrent MR detected beyond the first year did not result in subsequent mitral reoperation, but recurrent MR ≥2+ observed within the first year was associated with a 5-fold risk in subsequent mitral valve reoperation. There were 103 patients presenting with anterior leaflet prolapse, which was corrected with artificial neochordae or chordal transfer. Of all patients, recurrent MR of 2+ or higher occurred in 49 patients (5.7% of total) at a mean of 3.1 ± 2.5 years. The regurgitant jet was central in 16 and eccentric in 27. Of those with an eccentric regurgitant jet, 13 had an isolated anterior leaflet prolapse at initial presentation [10].

More recently, Ram et al. followed 760 patients who underwent surgical mitral valve repair clinically for a mean of 67 ± 47 months and with echocardiography for 50 ± 45 months [23]. Of these patients, 52 presented with anterior leaflet pathology. There were no significant differences of 5-year survival rates, mean ejection fraction, left ventricular end-diastolic diameter, and the rate of atrial fibrillation.

David et al. followed 1234 consecutive patients prospectively for a median of 13 years with periodical echocardiographic studies [24]. Of these patients, 127 presented with anterior leaflet prolapse. They did not compare mitral valve repair outcomes for those patients with anterior leaflet prolapse, posterior, or bileaflet prolapse. They reported that while the reoperation rate was low, a successful repair does not guarantee freedom from cardiac-related morbid events in long-term follow-up. Mild impairment in the left ventricular ejection fraction (LVEF) with the development of congestive heart failure (CHF) decreases the longitudinal survival rates. The leading cause of cardiac death was CHF, with most patients not being able to be helped with reoperation due to severe ventricular dysfunction. Additionally, there was a significant increase in new-onset atrial fibrillation postoperatively at 20 years after surgery. The increased risk of cardiac-related morbid events over time highlights the need for more data on longitudinal outcomes following surgical mitral valve repair, especially in comparison with new techniques and procedural innovations to provide patients with more comprehensive treatment options for degenerative MR.

### 2.2. Bileaflet Pathology

Bileaflet mitral valve repair has also been associated with lower valve repair rates [19,22]. Javadikasgari et al. reported 6153 patients who underwent primary isolated mitral valve repair for degenerative disease, comparing 3101 patients who underwent mitral valve repair for simple disease (posterior prolapse) versus 3052 patients who underwent mitral valve repair for complex disease (anterior or bileaflet prolapse) [20]. As described above, there were no significant differences in 20-year survival rates, but patients with complex MV pathologies have increased the recurrence of MR 10 years after repair. Overall, there is excellent time-related survival but lower repair durability in complex MR patients, and lifelong postoperative echocardiographic surveillance along with clinical follow-up is beneficial in this population.

Chan et al. reported 380 patients (of 855) presenting with bileaflet prolapse requiring surgical mitral valve repair. Late postoperative echocardiography identified all six patients who after repair, had initially presented with bileaflet prolapse, and the interval from surgery to the development of recurrent MR was 2.7 ± 2.1 years [10]. Ram et al. had 223 patients presenting with bileaflet pathologies and did not report significant differences in the early and intermediate outcomes following surgical mitral valve repair [23]. There remains a lack of multi-center and randomized long-term data as these are predominantly single center experiences.

### 2.3. Posterior Leaflet Pathology

Isolated posterior leaflet repair was seen as a protective factor from MR progression over time [23]. Tatum et al. followed 446 patients who underwent mitral valve repair for a mean follow-up of 5.5 ± 3.8 years. Postoperative echocardiograms were obtained in 75% of patients at a mean of 24.3 ± 13.7 months. The cumulative incidence of MR progression of 2+ or higher was 10.5%, 21.0%, and 35.8% at 3, 5, and 10 years, respectively. Overall surgical mitral valve repair had excellent outcomes, with patients undergoing isolated posterior leaflet repair demonstrating less recurrence of repair failure during clinical and echocardiographic follow-up [25].

Mohty et al. reported that posterior leaflet prolapse repair, compared to anterior leaflet prolapse repair, was associated with a significantly lower reoperation rate at 15 years (11% vs. 28%) [26]. Conversely, De Bonis and colleagues reported a similar 10-year freedom from reoperation for posterior leaflet prolapse compared with anterior leaflet prolapse [27]. There were also no significant differences in overall survival between both groups. However, echocardiographic follow-up was not available in 71.5% of patients with posterior leaflet prolapse, so reoperation was used as the primary indicator of mitral repair durability [27].

Brescia et al. conducted a propensity-matched analysis comparing anterior versus posterior mitral valve repair [28]. They found no difference in reoperation rates or long-term survival between posterior and anterior repair. This study was also limited in longitudinal echocardiographic follow-up due to the lack of routine echocardiograms on asymptomatic patients in practice.

In the Chan et al. population analysis of 855 patients, 372 patients presented with posterior leaflet prolapse [10]. It was typically treated with resection (triangular in 179 and quadrangular with sliding plasty in 256) and reconstruction with leaflet reapproximation, and 170 patients received chordal transfer. Ram et al. reported 485 patients presenting with posterior leaflet pathology requiring mitral valve repair, with no significant differences in the early and intermediate outcomes [23]. There was a longer clinical follow-up than echocardiographic follow-up.

### 2.4. Mitral Annular Calcification

Mitral annular calcification (MAC) also presents a technical challenge during surgical mitral valve repair with the increased risk of intra and postoperative complications [29,30,31,32]. Chan et al. reported long-term outcomes in 119 patients undergoing surgical mitral valve repair, of whom MAC was observed [29]. The risk factors for MAC were older age, female gender, severe renal dysfunction, and larger preoperative left atrial size. The 5-year actuarial survival following mitral valve repair was 88.1 ± 2.4%, which was not different between patients with and without MAC. The 5-year freedom from recurrent MR (≥2+) was 83.8 ± 6.8% for patients with MAC, not significantly different (*p* = 0.2) from 91.1 ± 2.4% in patients without MAC. Hence, MAC does not necessarily affect the durability of mitral valve repair based on 5-year follow-up data.

Overall, severe MR after the repair is rare, and those who required subsequent reoperation were more likely to have a recurrent MR of 2+ or higher within the first year after the operation, supporting that the rate of mitral valve repair failure is nonlinear [10]. There is also a lack of longitudinal objective assessments for patients after surgical mitral valve repair. This could be attributed to a lack of resources for personnel along with serial echocardiographic and clinical follow-up. Additionally, the cost of patient travel to testing serves as a barrier. Increasing long-term outcomes of follow-up provides data for better pre-operative decision making for both surgeons and patients.

### 2.5. Asymptomatic Patients

The 2020 American College of Cardiology/American Heart Association (ACC/AHA) guidelines recommend early surgery for asymptomatic patients with severe MR if the success rate of mitral valve repair is expected to exceed 95%, or if the left ventricular ejection fraction is ≤60%. However, some expert centers address MR in asymptomatic patients before the development of left ventricular dysfunction or enlargement, as asymptomatic patients with severe MR have a 20% 5-year mortality risk or 33% of a major cardiac event, including congestive heart failure within 5 years of diagnosis [33]. Chan and colleagues performed a longitudinal cohort study assessing 150 asymptomatic patients who underwent surgical mitral valve repair for primary MR that showed a 93.4% 5-year survival rate, and 94% of this cohort did not have recurrent MR ≥2+ at the 5-year follow-up [5]. Overall, this study shows that mitral valve repair is associated with favorable long-term outcomes following repair in patients with asymptomatic severe MR. Kang and colleagues conducted a propensity analysis of a large cohort of asymptomatic patients with severe MR and preserved left ventricular systolic function [34]. Early surgery, defined as elective surgery performed within 6 months of initial echocardiographic evaluation, was associated with significant long-term reductions in cardiac mortality and major cardiac events. Suri et al. used the Mitral Regurgitation International Database and included data of 1021 asymptomatic patients with flail mitral valve regurgitation that do not meet class I ACC/AHA guidelines for surgical mitral valve intervention [35]. Patients with early surgery, defined as surgery performed within 3 months from diagnosis, were found to have statistically significant higher long-term survival rates (86% vs. 69%). There was also a decreased long-term heart failure risk in the early surgery patient group. This data suggests that early surgery could provide a survival benefit in asymptomatic patients with flail leaflets.

### 2.6. Summary and Limitations

All of the studies above followed patients longitudinally after surgical mitral valve repair for primary MR and show the durability of surgical mitral valve repair irrespective of the specific leaflet pathology. Several studies highlight the value of surveillance echocardiographic follow-up in asymptomatic patients. However, a big limitation in the existing body of literature is that it is entirely based on observational studies, which cannot completely exclude selection bias at the level of statistical analysis. Additionally, the decision regarding which technique to use for the mitral valve repair is left to the discretion of the individual surgeon, which is an additional factor that may impact the outcomes. There is also a paucity of data on the long-term evolution of MAC in surgical mitral valve repair patients.

Many of the above studies are also single-center experiences. While this may limit heterogeneity within each study, taken together, there may be heterogeneity in the overall surgical practice. For instance, routine echocardiograms on asymptomatic patients following successful valve repair may not be uniformly performed. Overall, the observational nature of the included studies as well as heterogenous surgical and postoperative surveillance approaches to mitral valve repair poses some challenge in interpreting the long-term outcomes of the data in this population.

## 3. Minimally Invasive Mitral Valve Surgery and Catheter-Based Interventions

As cardiac surgery moves towards smaller incisions and faster recovery times, minimally invasive mitral valve surgery has been shown to be at least non-inferior to conventional mitral valve surgery via median sternotomy [36,37]. Eqbal et al. conducted a systematic review and meta-analysis of 117 studies comparing the outcomes of conventional versus minimally invasive mitral valve surgery, including robotic mitral valve surgery [37]. Minimally invasive approaches were associated with a shorter hospital length of stay with no increased morbidity or mortality. However, there was a lack of long-term follow-up on the data available in the included studies and the conclusions were limited based on the quality of the evidence available.

Jackson et al. compared 37 patients undergoing conventional mitral valve repair through median sternotomy versus 59 patients undergoing minimally invasive mitral valve surgery with a mean clinical follow-up of 6.3 years and a mean echocardiographical follow-up of 3.2 years [38]. There was a significantly higher 6-year mortality rate in patients who underwent conventional surgical repair (14%) compared with patients who underwent minimally invasive mitral valve repair (1.7%). There were no significant differences in re-intervention rates or time to re-do surgery. The mean left ventricular end-diastolic diameter reduction over time was similar in both cohorts. Overall, this is the first cohort study to show long-term outcomes of a lower mortality and the rate of recurrent MR in a minimally invasive mitral valve surgery cohort, and no difference between rates of re-operation or elevated mean mitral valve gradients >5 mmHg. However, the results are limited by the small number of patients and observational nature of the study.

Smaller cohort studies have shown no differences in 5-year survival rates and reintervention-free survival between conventional and minimally invasive groups, with minimally invasive mitral valve repair reducing ICU and hospital length of stay, readmission rates, and the need for blood transfusions. The durability of repair through long-term follow-up has also been equivocal between conventional and minimally invasive groups. Nonetheless, here is a lack of robust RCTs comparing the longitudinal outcomes between these approaches [39,40,41,42,43].

When comparing minimally invasive approaches, robotic versus nonrobotic mitral valve repair via right mini-thoracotomy, Zheng et al. showed that the mid-term survival was comparable with both techniques and a reduced hospital length of stay after robotic operations [44]. There is an overall scarcity of data on the long-term objective outcomes, such as survival and echocardiographical data of different minimally invasive approaches to mitral valve repair.

The emergence of transcatheter mitral valve interventions can provide high-risk patients with a better and safer option for management. Czarnecki et al. studied a cohort of 523 patients who underwent transcatheter mitral valve repair in Canada from 2011 to 2017 [45]. This population-based study showed a significant and sustained decrease in all-cause and heart failure related hospitalizations within 1 year after transcatheter mitral valve repair compared with prior to the intervention. As well, transcatheter mitral valve repair may be a viable treatment option for patients presenting with cardiogenic shock, who would not be candidates for surgery [46,47]. Nevertheless, the long-term outcomes following transcatheter mitral valve repair continue to be refined.

The included studies and systematic review and meta-analysis demonstrates short-term benefits of minimally invasive approaches to mitral valve repair associated with shorter ICU and hospital length of stay, as well as the readmission rates and need for blood transfusions. Some small cohort studies have also demonstrated equivocal long-term outcomes comparing conventional and minimally invasive groups. However, there is a limitation in the existing data in robust RCTs that compare conventional and minimally invasive approaches for patients with primary mitral regurgitation. Additionally, there is limited data on the longitudinal outcomes of patients following transcatheter mitral valve interventions. These novel interventions are shown to provide incredible value in certain patient populations in acute settings and there is a need for a further investigation into their role in the long-term.

## 4. Sex-Based Differences after Mitral Valve Repair

Few studies have evaluated the impact of sex on outcomes following the surgical repair of degenerative MR. The female gender is associated with increased adverse outcomes after mitral valve repair [48,49,50,51,52,53]. When analyzing a cohort of 1012 patients who underwent mitral valve repair of degenerative MR at the University of Ottawa Heart Institute, Ottawa, Ontario, Canada, the women in the cohort were older than the men and were less likely to present to surgery without symptoms [49]. Propensity analysis was conducted to adjust for differing preoperative variables, and while there were no significant differences in 10-year survival, women had worse left ventricular remodeling at a mean follow-up of 5.1 years. It is not fully understood why women have an increased risk of certain adverse outcomes following mitral valve repair. Women are more likely to present to surgery later, with echocardiographic markers suggestive of more advanced disease at the time of surgery [48,49,50,51,52,53]. Additionally, patients who present with more complex mitral valve disease and a higher prevalence of leaflet calcification are more likely to be female [48,49,50,51,52,53]. Given that current guidelines advising the management of asymptomatic patients with MR do not index to body surface area, women may be undergoing surgery at a point beyond optimal ventricle recovery [48,49,50,51,52,53]. There is a need for research and guidelines to address these management gaps to accommodate for a patient’s individual optimal recovery.

## 5. Discussion

Overall, surgical mitral valve repair is the gold standard therapy for patients with primary MR, and it is associated with a low risk for reoperation. While there exists many studies examining the long-term outcomes following surgical mitral valve repair in anterior, posterior, and bileaflet pathologies, there is a need for an ongoing improvement in patient follow-up and reporting. Additionally, research is needed to further refine strategies to minimize morbid events in the long-term. This applies to thoracoscopically and robotically assisted approaches in select patient sub-groups. While short term data have shown benefits in minimally invasive mitral valve repair for decreasing ICU and hospital lengths of stay, undoubtedly, long-term cohort data and randomized controlled trials would further refine treatment. This applies not only to surgery, but also to catheter-based interventions.

There is also a knowledge gap defining the differential outcomes of women regarding survival, repair rates, and left ventricular remodeling following surgical mitral valve repair. The current guidelines for primary MR management in asymptomatic patients do not index for body surface area and different sex-based anatomies and physiologies, which could be contributing to women undergoing surgery at a point beyond optimal ventricle recovery. Filling this gap may lend to changes in the current guidelines. Notwithstanding, the recent innovations to mitral valve therapy allow for a tailored approach to manage complex patients in the long-term.

## Figures and Tables

**Table 1:** Summary of studies evaluating longitudinal outcomes of patients undergoing mitral valve repair for degenerative mitral regurgitation and associated follow-up.

Study	Study Type	Number of Patients	Population (Anterior, Posterior, Bileaflet)	Mean Follow-Up Duration (Years)	Completeness of Follow-Up	Echocardiography at Follow-Up? (Y/N)
Badhwar 2012	Retrospective cohort, multicenter	14,604	Unspecified	5.9 ± 3.9	All patients followed, survival was 74.9% (10,934 of 14,604)	N
Chan 2016	Prospective cohort, single center	855	Anterior, posterior, and bileaflet	4.3 ± 3.5	All patients	Y (1 month, 3–6 months, and 12 months), mean echo follow-up 3.8 ± 3.2 years
Ram 2020	Retrospective cohort, single center	760	Anterior, posterior, and bileaflet	5.6 ± 3.9	35 (4.6%) patients died during follow-up; 16 during first 5 years after surgery	Y
Javadikasgari 2018	Retrospective cohort, single center	6153	Anterior or bileaflet (complex), posterior (simple)	Median 6 years	Unclear	Y with inconsistent f/u
Gillinov 2008	Retrospective cohort, single center	3544	Anterior, posterior	5.3 ± 4.8	20 patients lost to follow-up	N
David 2019	Prospective cohort, multicenter	1234	Anterior, posterior, and bileaflet	Median 13 (IQR 8, 34)	85% complete	Y with median of 11 years (IQR 6, 16), 65% complete
Tatum 2017	Retrospective cohort, single center	446	Anterior, posterior, and bileaflet	5.5 ± 3.8	All patients	Y in 334 patients (75%) at a mean of 24.3 ± 13.7 months
Mohty 2001	Retrospective cohort, single center	917	Anterior, posterior	7.7 ± 4.1	97% complete	Y performed in 815 patients at 3 ± 3 years
De Bonis 2006	Retrospective cohort, single center	851	Anterior, posterior	4.5 ± 3.12	100% in anterior leaflet group, 97.2% in posterior leaflet group	Y
Brescia 2021	Retrospective cohort, single center	1025	Anterior, posterior, bileaflet	7.3 (95% CI, 6.9–7.8)	All patients	N—no consistent long-term echocardiographic follow-up for all patients
Motazedian 2022	Retrospective cohort, single center	738	Unspecified	Unspecified, data reported at 5, 10, and 15 years	93% complete	N

IQR = interquartile range, CI = confidence interval.

## Data Availability

Not applicable.

## References

[1] De Bonis M., Alfieri O., Dalrymple-Hay M., Del Forno B., Dulguerov F., Dreyfus G. (2017). Mitral Valve Repair in Degenerative Mitral Regurgitation: State of the Art. Prog. Cardiovasc. Dis..

[2] El Sabbagh A., Reddy Y.N.V., Nishimura R.A. (2018). Mitral Valve Regurgitation in the Contemporary Era: Insights Into Diagnosis, Management, and Future Directions. JACC Cardiovasc. Imaging.

[3] Castillo J.G., Anyanwu A.C., Fuster V., Adams D.H. (2012). A near 100% repair rate for mitral valve prolapse is achievable in a reference center: Implications for future guidelines. J. Thorac. Cardiovasc. Surg..

[4] Lazam S., Vanoverschelde J.-L., Tribouilloy C., Grigioni F., Suri R.M., Avierinos J.-F., de Meester C., Barbieri A., Rusinaru D., Russo A. (2017). Twenty-Year Outcome After Mitral Repair Versus Replacement for Severe Degenerative Mitral Regurgitation. Circulation.

[5] Chan V., Ruel M., Elmistekawy E., Mesana T.G. (2015). Determinants of Left Ventricular Dysfunction After Repair of Chronic Asymptomatic Mitral Regurgitation. Ann. Thorac. Surg..

[6] Di Tommaso E., Rapetto F., Guida G.A., Zakkar M., Bruno V.D. (2021). Benefits of mitral valve repair over replacement in the elderly: A systematic review and meta-analysis. J. Card. Surg..

[7] Otto C.M., Nishimura R.A., Bonow R.O., Carabello B.A., Erwin J.P., Gentile F., Jneid H., Krieger E.V., Mack M., McLeod C. (2021). 2020 ACC/AHA Guideline for the Management of Patients With Valvular Heart Disease: A Report of the American College of Cardiology/American Heart Association Joint Committee on Clinical Practice Guidelines. Circulation.

[8] Motazedian F., Ostovar R., Hartrumpf M., Schröter F., Albes J.M. (2022). Every day mitral valve reconstruction: What has changed over the last 15 years?. PLoS ONE.

[9] Dumont E., Gillinov A.M., Blackstone E.H., Sabik J.F., Svensson L.G., Mihaljevic T., Houghtaling P.L., Lytle B.W. (2007). Reoperation After Mitral Valve Repair for Degenerative Disease. Ann. Thorac. Surg..

[10] Chan V., Elmistekawy E., Ruel M., Hynes M., Mesana T.G. (2016). How Does Mitral Valve Repair Fail in Patients With Prolapse?—Insights From Longitudinal Echocardiographic Follow-Up. Ann. Thorac. Surg..

[11] Zegdi R., Sleilaty G., Latrémouille C., Berrebi A., Carpentier A., Deloche A., Fabiani J.-N. (2008). Reoperation for Failure of Mitral Valve Repair in Degenerative Disease: A Single-Center Experience. Ann. Thorac. Surg..

[12] Al-Atassi T., Malas T., Mesana T., Chan V. (2014). Mitral valve interventions in heart failure. Curr. Opin. Cardiol..

[13] Patel J., Mandal K. (2020). Minimally invasive and transcatheter approaches for mitral valve surgery. Indian J. Thorac. Cardiovasc. Surg..

[14] Hensey M., Brown R.A., Lal S., Sathananthan J., Ye J., Cheung A., Blanke P., Leipsic J., Moss R., Boone R. (2021). Transcatheter Mitral Valve Replacement: An Update on Current Techniques, Technologies, and Future Directions. JACC Cardiovasc. Interv..

[15] Mack M., Carroll J.D., Thourani V., Vemulapalli S., Squiers J., Manandhar P., Deeb G.M., Batchelor W., Herrmann H.C., Cohen D.J. (2021). Transcatheter Mitral Valve Therapy in the United States: A Report From the STS-ACC TVT Registry. J. Am. Coll. Cardiol..

[16] Russo G., Gennari M., Gavazzoni M., Pedicino D., Pozzoli A., Taramasso M., Maisano F. (2021). Transcatheter Mitral Valve Implantation: Current Status and Future Perspectives. Circ. Cardiovasc. Interv..

[17] Shah M., Jorde U.P. (2019). Percutaneous Mitral Valve Interventions (Repair): Current Indications and Future Perspectives. Front. Cardiovasc. Med..

[18] Demir O.M., Bolland M., Curio J., Søndergaard L., Rodés-Cabau J., Redwood S., Prendergast B., Colombo A., Chau M., Latib A. (2021). Transcatheter Mitral Valve Replacement: Current Evidence and Concepts. Interv. Cardiol. Rev..

[19] Badhwar V., Peterson E.D., Jacobs J.P., He X., Brennan J.M., O’Brien S.M., Dokholyan R.S., George K.M., Bolling S.F., Shahian D.M. (2012). Longitudinal Outcome of Isolated Mitral Repair in Older Patients: Results From 14,604 Procedures Performed From 1991 to 2007. Ann. Thorac. Surg..

[20] Javadikasgari H., Mihaljevic T., Suri R.M., Svensson L.G., Navia J.L., Wang R.Z., Tappuni B., Lowry A.M., McCurry K.R., Blackstone E.H. (2018). Simple versus complex degenerative mitral valve disease. J. Thorac. Cardiovasc. Surg..

[21] Mick S., McCurry K., Navia J., Gillinov M. (2017). How long will my repair last, doctor? Additional data on the durability of mitral valve repair. J. Thorac. Cardiovasc. Surg..

[22] Gillinov A.M., Blackstone E.H., Alaulaqi A., Sabik J.F., Mihaljevic T., Svensson L.G., Houghtaling P.L., Salemi A., Johnston D.R., Lytle B.W. (2008). Outcomes After Repair of the Anterior Mitral Leaflet for Degenerative Disease. Ann. Thorac. Surg..

[23] Ram E., Schwammenthal E., Cohen H., Kogan A., Peled Y., Sternik L., Raanani E. (2020). Outcomes of Degenerative Mitral Valve Repair Surgery for Anterior, Posterior, and Bileaflet Pathology. Ann. Thorac. Surg..

[24] David T.E., David C.M., Tsang W., Lafreniere-Roula M., Manlhiot C. (2019). Long-Term Results of Mitral Valve Repair for Regurgitation due to Leaflet Prolapse. J. Am. Coll. Cardiol..

[25] Tatum J.M., Bowdish M.E., Mack W.J., Quinn A.M., Cohen R.G., Hackmann A.E., Barr M.L., Starnes V.A. (2017). Outcomes after mitral valve repair: A single-center 16-year experience. J. Thorac. Cardiovasc. Surg..

[26] Mohty D., Orszulak T.A., Schaff H.V., Avierinos J.-F., Tajik J.A., Enriquez-Sarano M. (2001). Very Long-Term Survival and Durability of Mitral Valve Repair for Mitral Valve Prolapse. Circulation.

[27] De Bonis M., Lorusso R., Lapenna E., Kassem S., De Cicco G., Torracca L., Maisano F., La Canna G., Alfieri O. (2006). Similar long-term results of mitral valve repair for anterior compared with posterior leaflet prolapse. J. Thorac. Cardiovasc. Surg..

[28] Brescia A.A., Watt T.M.F., Rosenbloom L.M., Murray S.L., Wu X., Romano M.A., Bolling S.F., Michigan Mitral Research Group (2021). Anterior versus posterior leaflet mitral valve repair: A propensity-matched analysis. J. Thorac. Cardiovasc. Surg..

[29] Chan V., Ruel M., Hynes M., Chaudry S., Mesana T.G. (2013). Impact of mitral annular calcification on early and late outcomes following mitral valve repair of myxomatous degeneration. Interact. Cardiovasc. Thorac. Surg..

[30] Carpentier A.F., Pellerin M., Fuzellier J.F., Relland J.Y. (1996). Extensive calcification of the mitral valve anulus: Pathology and surgical management. J. Thorac. Cardiovasc. Surg..

[31] MacVaugh H., Joyner C.R., Johnson J. (1971). Unusual complications during mitral valve replacement in the presence of calcification of the annulus. Ann. Thorac. Surg..

[32] Deniz H., Sokullu O., Sanioglu S., Sargin M., Ozay B., Ayoglu U., Aykut Aka S., Bilgen F. (2008). Risk factors for posterior ventricular rupture after mitral valve replacement: Results of 2560 patients. Eur. J. Cardio Thorac. Surg..

[33] Enriquez-Sarano M., Avierinos J.-F., Messika-Zeitoun D., Detaint D., Capps M., Nkomo V., Scott C., Schaff H.V., Tajik A.J. (2005). Quantitative Determinants of the Outcome of Asymptomatic Mitral Regurgitation. N. Engl. J. Med..

[34] (2014). Early Surgery Versus Conventional Treatment for Asymptomatic Severe Mitral Regurgitation: A Propensity Analysis. J. Am. Coll. Cardiol..

[35] Suri R.M., Vanoverschelde J.-L., Grigioni F., Schaff H.V., Tribouilloy C., Avierinos J.-F., Barbieri A., Pasquet A., Huebner M., Rusinaru D. (2013). Association Between Early Surgical Intervention vs Watchful Waiting and Outcomes for Mitral Regurgitation Due to Flail Mitral Valve Leaflets. JAMA.

[36] Missault S., Causenbroeck J.V., Vandewiele K., Czapla J., Philipsen T., François K., Bové T. (2020). Analysis of clinical outcome and postoperative organ function effects in a propensity-matched comparison between conventional and minimally invasive mitral valve surgery. J. Card. Surg..

[37] Eqbal A.J., Gupta S., Basha A., Qiu Y., Wu N., Rega F., Chu F.V., Belley-Cote E.P., Whitlock R.P. (2022). Minimally invasive mitral valve surgery versus conventional sternotomy mitral valve surgery: A systematic review and meta-analysis of 119 studies. J. Card. Surg..

[38] Jackson M., Sandhu M.S., Dong C., Bawamia B., Qureshi M., Khan K., Goodwin A., Kendall S., Hunter S., Graham R. (2018). Long-Term Outcomes Comparing Minimally Invasive Mitral Valve Repair versus Conventional Mitral Valve Surgery. World J. Cardiovasc. Surg..

[39] Olsthoorn J.R., Heuts S., Houterman S., Maessen J.G., Sardari Nia P., the Cardiothoracic Surgery Registration Committee of the Netherlands Heart Registration (2022). Effect of minimally invasive mitral valve surgery compared to sternotomy on short- and long-term outcomes: A retrospective multicentre interventional cohort study based on Netherlands Heart Registration. Eur. J. Cardiothorac. Surg..

[40] Sá M.P.B.O., Van den Eynde J., Cavalcanti L.R.P., Kadyraliev B., Enginoev S., Zhigalov K., Ruhparwar A., Weymann A., Dreyfus G. (2020). Mitral valve repair with minimally invasive approaches vs sternotomy: A meta-analysis of early and late results in randomized and matched observational studies. J. Card. Surg..

[41] Bowdish M.E., Elsayed R.S., Tatum J.M., Cohen R.G., Mack W.J., Abt B., Yin V., Barr M.L., Starnes V.A. (2021). Equivalent outcomes with minimally invasive and sternotomy mitral valve repair for degenerative mitral valve disease. J. Card. Surg..

[42] Huang H., Yan Q., Xie X., Zhou K., He B., Yang L. (2020). Early outcomes of mitral valvuloplasty by minimally invasive surgery or sternotomy. Asian Cardiovasc. Thorac. Ann..

[43] Takagi H., Hari Y., Nakashima K., Kuno T., Ando T., ALICE (All-Literature Investigation of Cardiovascular Evidence) Group (2020). Meta-analysis of propensity matched studies of robotic versus conventional mitral valve surgery. J. Cardiol..

[44] Zheng C.R., Mazur P., Arghami A., Jahanian S., Viehman J.K., King K.S., Dearani J.A., Daly R.C., Rowse P.G., Bagameri G. (2022). Robotic vs. minimally invasive mitral valve repair: A 5-year comparison of surgical outcomes. J. Card. Surg..

[45] Czarnecki A., Han L., Abuzeid W., Cantor W.J., Chan V., Cohen E.A., Cohen G.N., Fam N., Garg P., Hibbert B. (2021). Impact of Transcatheter Mitral Valve Repair on Preprocedural and Postprocedural Hospitalization Rates. JACC Cardiovasc. Interv..

[46] Jung R.G., Simard T., Kovach C., Flint K., Don C., Di Santo P., Adamo M., Branca L., Valentini F., Benito-González T. (2021). Transcatheter Mitral Valve Repair in Cardiogenic Shock and Mitral Regurgitation: A Patient-Level, Multicenter Analysis. JACC Cardiovasc. Interv..

[47] Chan V., Messika-Zeitoun D., Labinaz M., Hynes M., Nicholson D., Dryden A., Mesana T., Hibbert B. (2019). Percutaneous Mitral Repair as Salvage Therapy in Patients with Mitral Regurgitation and Refractory Cardiogenic Shock. Circ. Cardiovasc. Interv..

[48] Messika-Zeitoun D., Candolfi P., Enriquez-Sarano M., Burwash I.G., Chan V., Philippon J.-F., Toussaint J.-M., Verta P., Feldman T.E., Iung B. (2020). Presentation and outcomes of mitral valve surgery in France in the recent era: A nationwide perspective. Open Heart.

[49] Chan V., Chen L., Messika-Zeitoun D., Elmistekawy E., Ruel M., Mesana T. (2019). Is Late Left Ventricle Remodeling After Repair of Degenerative Mitral Regurgitation Worse in Women?. Ann. Thorac. Surg..

[50] Tran A., Ruel M., Chan V. (2015). Gender differences in outcomes following cardiac surgery: Implications for managing patients with mitral valve disease. Curr. Opin. Cardiol..

[51] Chan V., Messika-Zeitoun D., Labinaz M., Hynes M., Nicholson D., Dryden A., Mesana T., Hibbert B. (2021). Impact of sex on outcomes after percutaneous repair of functional mitral valve regurgitation. J. Card. Surg..

[52] Chan V., Elmistekawy E., Ruel M., Hynes M., Mesana T. (2016). Abstract 20352: How Durable is Repair of Degenerative Mitral Regurgitation in the Young?. Circulation.

[53] Chan V., Chen L., Elmistekawy E., Ruel M., Mesana T.G. (2016). Determinants of late outcomes in women undergoing mitral repair of myxomatous degeneration. Interact. Cardiovasc. Thorac. Surg..

